# Biomedical Signal Processing and Modeling Complexity of Living Systems 2013

**DOI:** 10.1155/2013/173469

**Published:** 2013-11-20

**Authors:** Carlo Cattani, Radu Badea, Sheng-yong Chen, Maria Crisan

**Affiliations:** ^1^Department of Mathematics, University of Salerno, Via Ponte Don Melillo, 84084 Fisciano (SA), Italy; ^2^Department of Clinical Imaging Ultrasound, “Iuliu Hatieganu” University of Medicine and Pharmacy, 400000 Cluj-Napoca, Romania; ^3^College of Computer Science & Technology, Zhejiang University of Technology, Hangzhou 310023, China; ^4^Department of Histology, “Iuliu Hatieganu” University of Medicine and Pharmacy, 400000 Cluj-Napoca, Romania

Biomedical signal processing aims to provide significant insights into the analysis of the information flows from physiological signals. As such, it can be understood as a specific interdisciplinary scientific discipline. In fact, biomedical signals extract information from complex biological models thus proposing challenging mathematical problems, whose solution has to be interpreted from a biological point of view. The focus of this special issue is the mathematical analysis and modeling of time series in living systems and biomedical signals. The main steps of the biomedical signals processing are as follows.

1. Signal processing of biological data implies many different interesting problems dealing with signal acquisition, sampling, and quantization. The noise reduction and similar problems as image enhancement are a fundamental step in order to avoid significant errors in the analysis of data. Feature extraction is the most important part of the analysis of biological signals because of the importance which is clinically given to even the smallest singularity of the image (signal).
2. Information flows from signals imply the modeling and analysis of spatial structures, self-organization, environmental interaction, behavior, and development. Usually this is related to the complexity analysis in the sense that the information flows come from complex systems so that signals show typical features, such as randomness, nowhere differentiability, fractal behavior, and self-similarity which characterize complex systems. As a consequence typical parameters of complexity such as entropy, power spectrum, randomness, and multifractality play a fundamental role, because their values can be used to detect the emergence of clinical pathologies.
3. Physiological signals usually come as 1D time series or 2D images. The most known biosignals are based on sounds (ultrasounds), electromagnetic pulses (ECG, EEG, and MRI), radiation (X-ray and CT), images (microscopy), and many others. The clinical signal understanding of them follows from the correct, from a mathematical point of view, interpretation of the signal.
4. Physiological signals are detected and measured by modern biomedical devices. Among others, one of the main problems is to optimize both the investigation methods and the device performances.

The papers selected for this special issue represent a good panel in recent challenges. They represent some of the most recent advances in many different clinical investigations devoted to the analysis of complexity in living systems, like, for example, network science, dynamical systems theory, dynamical complexity, pattern analysis, implementation, and algorithms. They cannot be exhaustive because of the rapid growing both of mathematical methods of signal analysis and of the technical performances of devices. However they aim to offer a wide introduction on a multidisciplinary discipline and to give some of the more interesting and original solution of challenging problems. Among them the most fascinating is to understanding of the biological structure and organization, the intracellular exchange of information, the localization of information in cell nuclei, and in particular the unrevealing of the mathematical information (functionally related) content in DNA.

This special issue contains 23 papers. In the category of modeling dynamical complexity, L.-P. Tian et al. make complex analysis and parameter estimation of dynamic metabolic systems. M. Adib and E. Cretu present wavelet-based artifact identification and separation technique for EEG signals during galvanic vestibular stimulation. X. Wu and N. Wu use thresholded two-phase test sample representation for outlier rejection in biological recognition. Z. Ma et al. propose nonlinear Radon transform using Zernike moment for shape analysis. C.-Y. Liou et al. study structural complexity of DNA sequence. M. Li et al. investigate heavy-tailed prediction error in predicting biomedical signals of 1/f noise type. X. Wang et al. propose reliable RANSAC using a novel preprocessing model. J. Zheng et al. give fast discriminative stochastic neighbor embedding analysis.

In the category of methods for analysis of dynamical complexity, R. Schiavetti and G. Sannino give in vitro evaluation of ferrule effect and depth of post insertion on fracture resistance of fiber posts. G. Sannino and G. Vairo make comparative evaluation of osseointegrated dental implants based on platform-switching concept and find influence of diameter, length, thread shape, and in-bone positioning depth on stress-based performance. H.-T. Wu et al. use multiscale cross-approximate entropy analysis as a measure of complexity among the aged and diabetic. T. Kauppi et al. construct benchmark databases and protocols for medical image analysis with diabetic retinopathy. B. Zhu et al. present a novel automatic detection system for ECG arrhythmias using maximum margin clustering with an immune evolutionary algorithm. Y.-S. Juang et al. study optimization and implementation of scaling-free CORDIC-based direct digital frequency synthesizer for body care area network systems. Z. Bian et al. find the effect of Pilates training on alpha rhythm.

In the category of biomedical signal analysis, A. F. Badea et al. give fractal analysis of elastographic images for automatic detection of diffuse diseases of salivary glands. Q. Guan et al. present Bayes clustering and structural support vector machines for segmentation of carotid artery plaques in multicontrast MRI. J. Zhang et al. present self-adaptive image reconstruction inspired by insect compound eye mechanism. X. Zheng et al. improve spatial adaptivity of nonlocal means in low-dosed CT imaging using pointwise fractal dimension. N. Wu et al. study three-dimensional identification of microorganisms using a digital holographic microscope. Y. Tang et al. propose a computational approach to seasonal changes of living leaves. L. Lin et al. study plane-based sampling for a ray casting algorithm in sequential medical images. Y.-S. Juang et al. propose a rate-distortion-based merging algorithm for compressed image segmentation.

As already mentioned, the topics and papers are not an exhaustive representation of the area of biomedical signal processing and modeling complexity of living systems. However we believe that we have succeeded to collect some of the most significant papers in this area aiming to improve the scientific debate in the modern interdisciplinary field of biomedical signal processing.

